# Parents of young people with self-harm or suicidal behaviour who seek help – a psychosocial profile

**DOI:** 10.1186/1753-2000-7-13

**Published:** 2013-04-23

**Authors:** Sophia Morgan, Eóin Rickard, Martha Noone, Carole Boylan, Andreé Carthy, Sinead Crowley, John Butler, Suzanne Guerin, Carol Fitzpatrick

**Affiliations:** 1 The Children’s University Hospital, Temple Street, Dublin, Ireland; 2 University College Dublin, Dublin, Ireland

**Keywords:** Deliberate self-harm, Parents, Help-seeking, Adolescents, Suicidal behaviour, Parental well-being, Group support programme

## Abstract

**Background:**

Deliberate Self-Harm (DSH) is a common problem among children and adolescents in clinical and community populations, and there is a considerable amount of literature investigating factors associated with DSH risk and the effects of DSH on the child. However, there is a dearth of research examining the impact of DSH on parents, and there are few support programmes targeted at this population. This cross-sectional study examines the profile of a sample of parents of young people with DSH who participated in a support programme (Supporting Parents and Carers of young people with self-harm: the SPACE programme), with the goal of investigating pre-test parental well-being, family communication, parental satisfaction, perceived parental social support, and child strengths and difficulties.

**Methods:**

Participants were 130 parents who attended the SPACE programme between 2009 and 2012, and who completed six questionnaires at baseline: the General Health Questionnaire-12, Strengths and Difficulties Questionnaire, Kansas Parenting Satisfaction Scale, General Functioning Scale of the McMaster Family Assessment Device, Multidimensional Scale of Perceived Social Support, and a demographic questionnaire.

**Results:**

The majority of parents met criteria for minor psychological distress (86%) and rated the quantity and severity of their children’s difficulties as being in the abnormally high range (74%) at baseline. A majority of participants (61%) rated their perceived social support as being poor. Lower parental well-being was significantly correlated with poorer family communication, poorer parenting satisfaction, and a greater number of difficulties for the child. Perceived social support was not significantly correlated with parental well-being. Parents whose children were not attending school at baseline had significantly lower well-being scores than those whose children were. Parents whose children had received a formal diagnosis of a mental health disorder also had significantly lower well-being scores than those whose children had not.

**Conclusions:**

Parents of young people with DSH behaviours face considerable emotional and practical challenges; they have low levels of well-being, parenting satisfaction, social support, and experience poor family communication. Given the importance of parental support for young people with DSH behaviours, consideration should be given to the need for individual or group support for such parents.

## Background

Deliberate self harm (DSH) is a common problem among adolescents in both community and clinical samples [1-4]. This paper uses Hawton’s definition of DSH as ‘a non-fatal act in which an individual deliberately intended to cause self-harm through injury, ingestion of a substance in excess of the prescribed or therapeutic dose, ingestion of an illicit/recreational drug that was an act the individual regarded as self-harm or ingestion of a non-ingestible substance or object’ [5]. DSH covers a spectrum of behaviours, from an act of minor self injury to reduce emotional pain at one end of the spectrum, to attempted suicide at the other. While most young people who have self-harmed do not die by suicide, DSH is a risk factor for suicide in the years ahead [6,7], with over 40% of young people who die by suicide having a history of DSH [8]. Most young people with DSH do not present to medical services [1,2], but for those who do, an opportunity is provided for intervention which may reduce risk of future suicide.

Young people who engage in DSH, whether in clinical or community studies, report high levels of depression, anxiety, and relationship stresses, including family conflict [9,10]. Communication problems and family relationship difficulties in particular have been found to be associated with DSH [11,12]. A study comparing 52 adolescents who had presented to Accident and Emergency departments following DSH with 52 hospital-based controls with no history of DSH showed a strong association between the absence of a family confidante and adolescent self harm [13]. The authors suggested that poor communication within the family may lead the young person to feel socially isolated, and their problems to appear insurmountable, with DSH being perceived as their only option. Having a good relationship with parents has been reported as being a protective factor against suicidal behaviour and suicide in adolescents [14], but research in this area is sparse.

It is not possible to be clear about the nature of the association between family communication/relationship problems and adolescent DSH, as few prospective studies have been carried out in relation to this topic. Previous research has, however, found that poorer family functioning results from the occurrence of depression in adolescents, and that impairments to functioning may persist beyond six months after the depressive episode [15]. More specifically, it has been postulated that DSH behaviours have a ‘ripple effect’ on families [16,17]. A qualitative study by Raphael et al. [17] involved in-depth face to face interviews with parents of young people who had self-harmed, and gives particular insight into the emotional and practical challenges faced by parents in response to DSH activity. These parents reported that self-harm by their son/daughter was extremely traumatic for them, leading to feelings of helplessness, anger, grief, guilt, and failure. Participants voiced concerns about their ability to cope as parents when their young person was discharged from hospital, and questioned their own parenting skills and competence. In some cases, parents argued with one another as to how to manage the child’s DSH and try to prevent future incidents, or left their jobs in order to support the child. Some participants also reported experiencing somatic and psychological symptoms following the initial DSH incident (e.g., depression, insomnia), as well as interruption to their usual routines (e.g., being unable to go to work). Furthermore, participants reported a perceived lack of information and support for themselves from health services, leading to increased feelings of hopelessness and confusion.

These findings were echoed by those of a focus group study for parents and carers of young people with DSH who attended the Children’s University Hospital, Temple Street, Dublin [18]. The participants described the damaging effect of the DSH on their relationship with their adolescent. One parent said ‘*your trust is gone and it’s difficult to build that up with them again’*[18]. Another qualitative interview study with 12 parents described them as having ‘a strong and lasting emotional reaction’ to their young person’s DSH [19]. These parents reported that they were ‘walking on eggshells’, and having marked difficulties setting limits and maintaining boundaries with their young people.

Previous research examining the broader effects of adolescent mental health disorders (covering a wide range of difficulties, including mood disorder, schizophrenia, and obsessive-compulsive disorder) has described the subsequent difficulties encountered by parents as ‘caregiver burden’ [20-22]. This concept includes two aspects, the first being ‘subjective burden’, which relates to the parent’s own perception of the challenges that they are confronted with, and varies according to factors such as child and parental gender, socioeconomic status, and the child’s psychiatric condition and symptoms. The second, ‘objective burden’, refers to disruption to the structure of family life, such as reductions in leisure time, increased financial strain, deteriorating communication and social relations within the family, and changes in household routines [20,22-24]. Given the nature of the aforementioned emotional issues and practical difficulties that arise as a result of DSH, it is possible that the concept of caregiver burden may be applicable to parents whose child has experienced DSH, regardless of their child’s specific psychiatric diagnosis. In view of the above evidence, it is likely that the association between family communication and relationship difficulties and adolescent DSH is a complex and circular one, and that family relationships can have both detrimental and protective roles.

In 2006, a support programme for parents and carers of young people with DSH was developed by the DSH Team in the Children’s University Hospital, Temple Street. It is called the SPACE programme, and was developed in response to requests for Support by Parents And Carers of young people with DSH who had presented to the Accident & Emergency Department of the hospital. The programme was developed with input from parents, who advised on its format and content [18]. It is an eight week group programme, run for one and a half hours on one evening per week, which is both supportive and psycho-educational, and covers areas deemed important by the focus group parents, such as family communication, skills for parenting adolescents, and information about mental health difficulties in young people. The programme aims to provide support to parents to enable them to support their young person. It is run by two facilitators who are members of the DSH Team in Children’s University Hospital, Temple Street. The programme has been evaluated in a non-controlled pilot study, and appears to be effective in improving parents’ feelings of well-being, improving their satisfaction in their parenting role, and improving family communication [25]. It is currently being evaluated using a randomised controlled trial (RCT).

The aim of this research is to present a cross-sectional, pre-test demographic and psychosocial profile of the parents who participated in the evaluation of the SPACE programme, focusing on parental well-being, family communication, parental satisfaction, perceived social support, the child’s strengths and difficulties, as well as a number of adult and child characteristics. Potential relationships between these factors will also be examined.

## Method

### Participants

Study participants were parents who attended an Introductory Evening about SPACE, with a view to taking part in the next programme. Introductory Evenings were held in the week preceding each ‘run’ of the SPACE Programme, which took place three times per year from 2009 to 2012, and was run in a Dublin city centre hotel. In 2009, all parents were referred to the programme from the Child and Adolescent Mental Health Service or Family Support Service which their young person was attending. Due to the provision of additional funding in 2010, ‘11, and ‘12, the programme was broadened to include any parent, without referral, who was concerned about self-harm or suicidal behaviour in their young person under the age of 18 years. Information about the programme had been provided to family doctors, social services departments, and accident and emergency departments, and it had received publicity in national and local media. The only specified inclusion factors were that participants were a parent of a young person under the age of 18 where there were concerns about self-harm or suicidal behaviour, and that they gave their informed consent to participate in the study. There were no exclusion factors.

At each Introductory Evening, SPACE programme facilitators gave information pertaining to the background, aims, structure, and content of the programme. Use of the RCT to evaluate the programme was also explained to potential participants, whereby parents who consented to participate would be randomly allocated to either the programme starting the following week or to the subsequent programme, starting approximately five months later. Parents who consented to take part were asked to complete a socio-demographic questionnaire (see Additional file 1), and the five psychometric measures outlined below.

### Measures

#### The General Health Questionnaire (GHQ-12)

This is a widely used self-report screening tool used for the assessment of mental well-being [26]. It is a measure of common mental health problems across the domains of depression, anxiety, somatic symptoms, and social withdrawal. It has well established reliability and validity and has been shown to have internal consistency reliability coefficients of 0.82 to 0.86 in most studies [26,27]. The GHQ format was utilised for scoring of the measure in the present study (i.e., responses were scored as 0, 0, 1 and 1, respectively), as this format is recommended by the authors for the detection of cases, as opposed to comparing degrees of disorder. Using this scoring method, a score of 3 or higher indicates probable ‘caseness’.

#### The Kansas Parenting Satisfaction Scale (KPS)

This is a 3-item self-report measure designed to assess parent satisfaction with themselves as a parent, satisfaction with the behaviour of their children, and satisfaction with their relationship with their children. Higher scores on the KPS indicate higher levels of parenting satisfaction. The scale is reported to have good concurrent validity - significant correlations have been found with the Kansas Marital Satisfaction Scale and the Rosenberg Self Esteem Scale (0.23 to 0.55) [28].

#### General functioning scale of the McMaster Family Assessment Device (FAD)

The communication subscale of this assessment tool was utilised, and it is a reliable and valid 6-item self-report measure of the respondent’s perception of how well their family communicates with each other. Lower scores are indicative of healthier functioning [29-31].

#### Multidimensional Scale of Perceived Social Support (MSPSS)

This is a reliable and valid twelve item self-report measure of the adequacy of support from family, friends and significant others, as perceived by the individual. It provides a total score and three subscale scores. Those in the upper third of range are highly supported, and those in the lower third poorly supported [32,33].

#### The Strengths and Difficulties Questionnaire (SDQ)

This is a brief behavioural screening questionnaire for 3 to 16 year olds [34]. It consists of five subscales – emotional symptoms, conduct problems, hyperactivity, peer relationship problems and pro-social behaviour. All subscales except the pro-social behaviour subscale are added together to generate a total difficulties score. The SDQ subscales have a mean internal consistency reliability coefficient of 0.71, a mean test-retest reliability coefficient over six months of 0.62 and demonstrate good criterion validity for predicting psychological disorders [35]. A score of 17 or above is in the abnormal or ‘clinical’ range.

### Statistical analysis

SPSS version 20 was used for statistical analyses. Descriptive statistics were used to describe the demographic variables and include means, standard deviations and frequencies. The sample size varied slightly across the analyses due to instances of missing data. The relationships between scores on the GHQ and scores on each of the 4 other questionnaires were analysed using correlation analyses (Kendall’s Tau). Mann–Whitney U tests were used to examine differences in GHQ-12 scores between participant groups on a number of variables (e.g., gender, marital status).

### Ethics

Ethical approval for the study was granted by the Research Ethics Committee of the Children’s University Hospital, Temple Street, Dublin.

## Results

### SPACE participant demographics

Descriptive statistics for all measures administered to participants at baseline can be seen in Table 1, and caseness ranges for the GHQ-12, SDQ, and MSPSS can be seen in Table 2. One-hundred-and-thirty parents participated in the present study, with 82 (63%) of these being female and 48 (37%) male. Of these individuals, 49 attended the SPACE programme alone, while 81 stated that they attended with a partner (that this figure is an odd number most likely results from one parent in a couple not consenting to take part in the study, withdrawals, or the question being incorrectly answered by a participant). Sixty-nine parents (53%) were referred to the programme by a mental health service, while 61 (47%) attended as a result of being made aware of the programme by advertising in the community.

**Table 1:** Minimum and maximum scores, means, and standard deviations for measures administered to participants at baseline

**Measure**	***n***	**Minimum score**	**Maximum score**	***M***	***SD***
GHQ-12	129	0	12	7.76	3.79
FAD – Communication subscale	129	9	23	14.95	2.95
KPS	130	3	19	10.40	3.83
SDQ	125	5	35	20.78	6.75
MSPSS	128	12	84	58.60	16.86

**Table 2:** Caseness ranges for parental well-being, parental perceived social support, and child strengths and difficulties

**Variable**	***n***	**Falls within caseness range**	
		**Yes**	**No**
Well-being; (GHQ-12 score ≥ 3 = distress)	129	111 (86 %)	18 (14 %)
Social support; (MSPSS score ≤ 65 = poorly supported)	128	78 (61 %)	50 (39 %)
Child strengths and difficulties; (SDQ score ≥ 17 = abnormal)	125	93 (74 %)	32 (26 %)

Note: Total number of cases for SDQ relates to number of parents, as SDQ is subjective and two parents may rate the same child differently. No caseness ranges are available for Parental Satisfaction or Family Communication.

From Table 2, it can be seen that a sizeable majority of participants met the criteria for minor psychological distress (GHQ-12) and rated the number and severity of their children’s difficulties as being in the abnormal range (SDQ). A majority of participants also rated their perceived social support (MSPSS) as being poor.

Despite the SPACE programme being open to participation from both parents and full-time carers of children who have experienced issues with deliberate self-harm, the present sample focuses on data of parents only (*n* = 130). This approach was taken as the needs and experiences of parents differ from those of carers (e.g., carers may have already received training in dealing with mental health issues), and they comprised a sizeable majority of the participants overall; parents comprised 88% of the total sample (*N* = 147), with 12% (*n* = 17) of this total being carers. Demographic information for parents relating to their marital status can be seen in Table 3.

**Table 3:** Demographic information relating to parents’ marital status

	**Marital status (%)**						
	***n***	**Married**	**Living with partner**	**Single**	**Separated**	**Divorced**	**Widowed**
Total	130 (100)	106 (82)	5 (4)	4 (3)	11 (8)	3 (2)	1 (1)
Male	48 (37)	43 (90)	1 (2)	1 (2)	2 (4)	1 (2)	0 (0)
Female	82 (63)	63 (77)	4 (5)	3 (4)	9 (11)	2 (2)	1 (1)

In terms of participants’ socioeconomic status, the SPACE programme attracted a relatively balanced mix of individuals from all strata of society. Forty-six participants (35%) identified themselves as being in professional, managerial and technical, or non-manual employment, while 39 (30%) identified themselves as being in skilled, semi-skilled, or unskilled manual employment. Seventeen participants (13%) identified themselves as being homemakers, 7 participants (6%) identified themselves as being unemployed, and data were missing for 21 (16%) participants.

Information pertaining to the mental health of participants at baseline can be seen in Table 4; a majority of parents reported having never had a mental health disorder, while depression was the condition most frequently reported by participants who had experienced a mental health disorder at some point in their lives. Reported self-harm behaviour among parents was relatively low, at 6%.

**Table 4:** Demographic information on mental health issues experienced by parents

**Variable**	***n***	**%**
Parent mental health	130	100
No history of mental health issues	71	55
Depression	27	21
Anxiety	11	8
Alcohol problems	1	1
Two or more issues	19	14
Missing data	1	1
Parent history of self-harm	130	100
Yes	8	6
No	120	92
Missing data	2	2

### Child demographics

Parents reported that their child with DSH behaviours (*N* = 99; parents’ reports were checked and combined in cases where two parents reported on the same child) was female in 62% (*n* = 61) of cases and male in 38% (*n* = 38), with a mean age of 14.62 years (*SD* = 2.307), and ages ranging from 5–18 years. A majority of these children were attending school at baseline (*n* = 83; 84%), with 15 children (15%) not attending school, and data missing for 1 case (1%).

**Figure 1:**
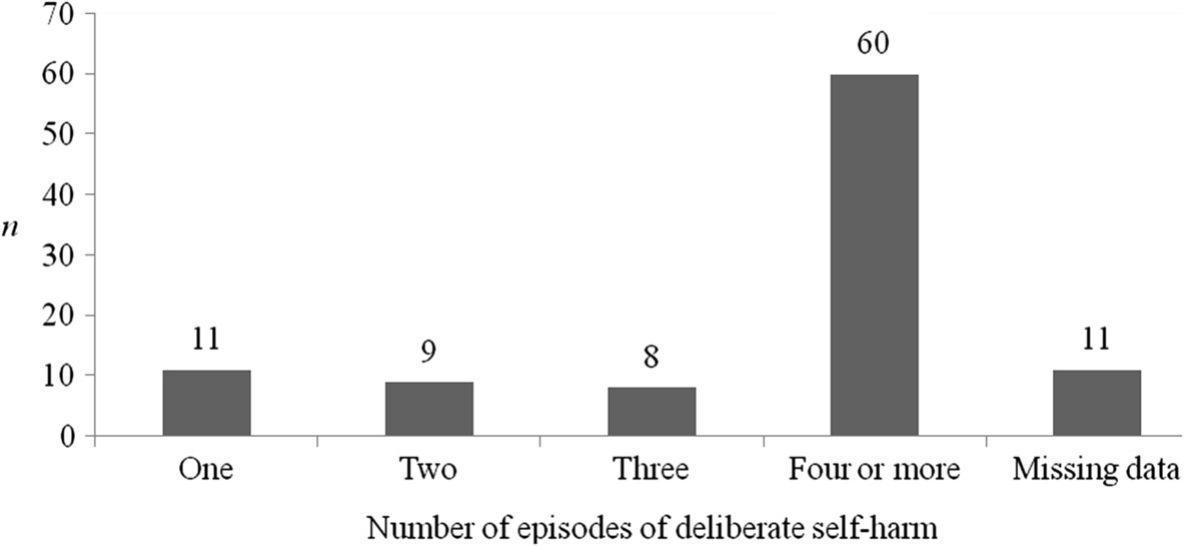
Number of episodes of deliberate self-harm experienced by the child (N = 99), as reported by parents.

**Figure 2:**
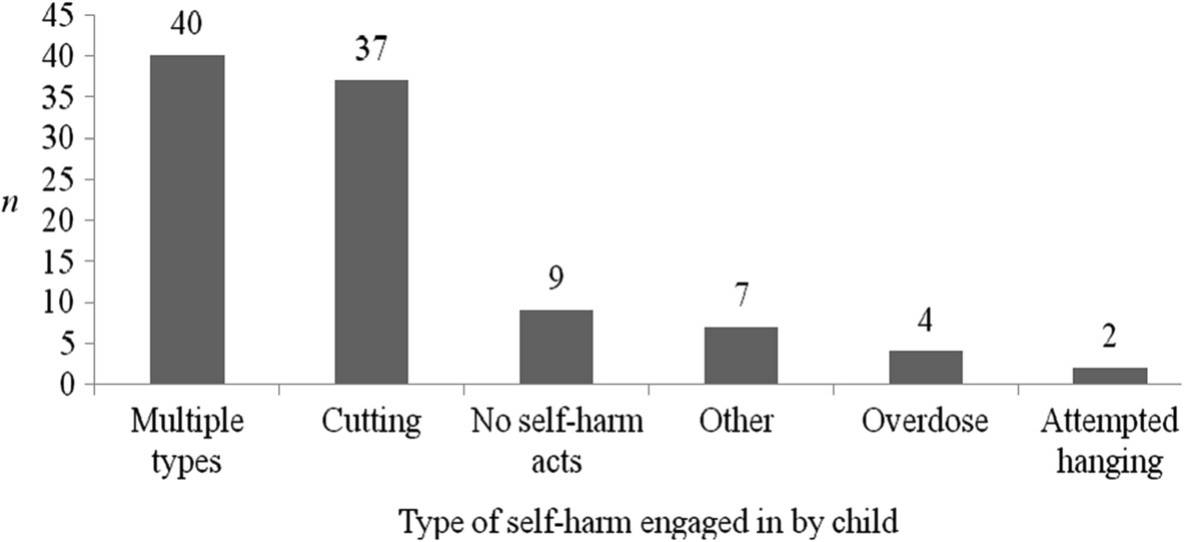
Type of deliberate self-harm engaged in by the child (N = 99), as reported by parents.

**Figure 3:**
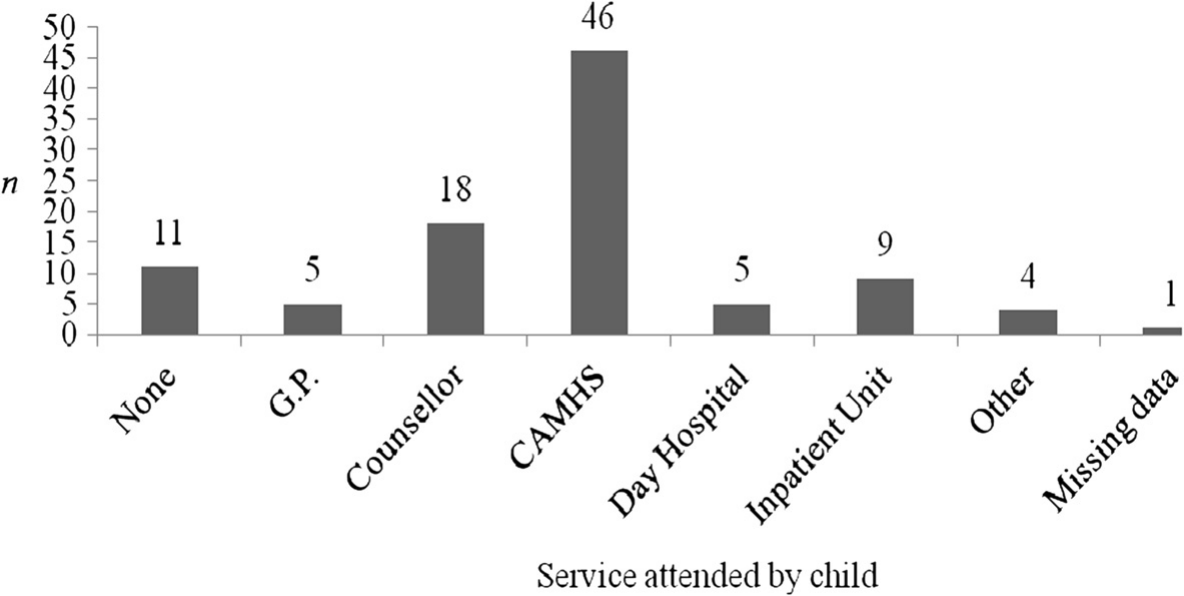
Type of service attended by child experiencing self-harm issues (N =99, as reported by parents).

Information on whether the child has a formal diagnosis of a mental disorder and on DSH-specific behaviours engaged in by children (as reported by parents) can be seen in Table 5, and Figures 1 and 2. These figures indicate that the majority of parents attending SPACE were coping with repeated episodes of DSH in the child over time, and with multiple different methods of DSH also. From these figures, it can be ascertained that, in the majority of cases, DSH was a severe and recurrent issue for the adolescents whose parents took part in SPACE, indicating that they are an at-risk group. The type of mental health care service being attended by the child can be seen in Figure 3.

**Table 5:** Parental report of formal diagnosis of children with DSH behaviours, and nature of DSH behaviours (N = 99)

**Variable**	***n***	**%**
Diagnosis		
No formal diagnosis	38	39
Depression	28	28
Behavioural/conduct problem	3	3
ADHD	3	3
Substance misuse (i.e., drugs, alcohol)	1	1
Multiple diagnoses	25	25
Missing data	1	1
Child has experienced thoughts of DSH		
Yes	84	85
No	15	15
Child has experienced a DSH episode		
Yes	89	90
No	10	10

Note: There is an overlap between children who experienced DSH thoughts and episodes, i.e., all children experienced either thoughts or an episode of DSH, and some experienced both. A lack of DSH thoughts combined with a DSH episode is considered to be an impulsive episode, with no previous indication of intent from the child.

### Parental well-being

#### Correlations

As outlined previously, parental well-being was assessed using the GHQ-12. GHQ data was found to violate the assumption of normality required for the use of parametric measures, as evidenced by a significant Kolmogorov-Smirnov statistic (.153, *p* < .05). It was not possible to use mathematical transformation with the GHQ data, therefore correlation and group difference analyses are performed using non-parametric tests.

The relationship between parental well-being and a number of purportedly associated variables was assessed using Kendall’s Tau correlation coefficient (due to the relatively large number of tied ranks in the data) and Cohen’s criteria to calculate effect size [36]; these relationships can be seen in Table 6.

**Table 6:** Correlations of parental well-being at baseline with reported child difficulties, and with parental social support, parenting satisfaction and family communication

**Variable**	***n***	**Kendall’s Tau**	***p***
Child’s strengths and difficulties (SDQ total)	125	.174	.007*
Perceived social support (MSPSS)	128	-.046	.470
Parenting satisfaction (KPS)	129	-.404	.000*
Family communication (FAD subscale)	128	.142	.030**

Notes: *Correlation significant at p < .01 level. **Correlation significant at p < .05 level. A high score on the GHQ (i.e., ≥ 3) indicates poorer well-being. A high score on the SDQ (i.e., ≥ 17) indicates abnormal functioning. A high score on the MSPSS (upper third of scores) indicates high support. A high score on the KPS indicates higher satisfaction. A high score on the FAD indicates poorer communication functioning.

There was a small positive correlation between parental well-being and reported child difficulties, indicating that poorer parental well-being is associated with a higher number of reported difficulties for the child. There was a strong, negative correlation between parental well-being and parenting satisfaction, which indicates that poorer parental well-being is related to lower levels of parenting satisfaction. A small, positive correlation was observed between parental well-being and family communication, whereby poorer parental well-being was associated with poorer family communication. The relationship between parental well-being and parental perceived social support was found not to be statistically significant.

#### Parental well-being: group differences

A series of Mann–Whitney U tests were performed to examine group differences for a number of variables, relating to both parents and children, in relation to parental well-being. Variables relating to parents are examined first, followed by those relating to children.

The first variables examined were parental gender and whether parents attended SPACE alone or as part of a couple. As can be seen in Table 7, a statistically significant group difference in well-being levels was identified only for gender, with a small effect size [36], whereby females’ reported levels of well-being were lower than those of males. There was no statistically significant difference between parents who attended SPACE alone and those who attended with a partner.

**Table 7:** Mann–Whitney U tests investigating group differences for gender and how SPACE was attended, in relation to parental well-being

	**Mann–Whitney U**			
	**Gender**		**How SPACE was attended**	
Statistic	*Male*	*Female*	*Alone*	*With partner*
*N*	48	81	48	81
Median	7.5	9	9	8
Mean ranks	56.72	69.91	66.90	63.88
Sum of ranks	2722.5	5662.5	3211	5174
*U*	1546.5		1853	
Z	−1.958		-.448	
*P*	.05		.654	
*R*	.172		.0394	

As evidenced from Table 8, there was no statistically significant difference in well-being levels between parents who had a history of experiencing mental health disorders and those who did not (although the difference between these groups approached statistical significance, and thus may be of clinical significance), and parents who had a history of DSH behaviours and those who did not.

**Table 8:** Mann–Whitney U tests investigating group differences for parental mental health history and history of DSH, in relation to parental well-being

	**Mann–Whitney U**			
	**Parent has experienced a mental health disorder**		**Parent has experienced DSH behaviours**	
Statistic	*Yes*	*No*	*Yes*	*No*
*n*	58	70	8	119
Median	9	7.5	11	8
Mean ranks	71.24	58.91	83.44	62.69
Sum of ranks	4132	4124	667.5	7460.5
*U*	1639		320.5	
*z*	−1.892		−1.560	
*p*	.058		.119	
*r*	.167		.137	

Results for Mann–Whitney U tests performed to investigate variables relating to the child who engaged in DSH behaviours can be seen in Table 9. Analyses here include couples, both of whom have reported on the same child. However, as the dependent variable in these analyses is parental well-being, and as it is the effect of the child’s characteristics on each individual parent that is being investigated, these cases are not considered to be duplicates.

**Table 9:** Mann–Whitney U tests investigating group differences for child school attendance and diagnosis of a mental health disorder, in relation to parental well-being

	**Mann–Whitney U**			
	**Child was attending school at time 1**		**Child has had formal diagnosis of mental health disorder**	
Statistic	*Yes*	*No*	*Yes*	*No*
*n*	107	21	80	48
Median	8	11	9	6
Mean ranks	61.40	80.31	70.02	55.30
Sum of ranks	6569.5	1686.5	5601.5	2654.5
*U*	791.5		1478.5	
*z*	−2.159		−2.197	
*p*	.031		.028	
*r*	.191		.194	

A statistically significant difference in parental well-being scores was identified for children attending or not attending school at baseline, and between children who had received a formal diagnosis of a mental health disorder and those who had not, with a small effect size for both [36]. Parental well-being was lower if the child was not attending school at baseline, and was lower if the child had received a formal diagnosis of a mental health disorder.

### Summary

The majority of parents met criteria for minor psychological distress (86%) and rated the quantity and severity of their children’s difficulties as being in the abnormally high range (74%) at baseline. A majority of participants (61%) rated their perceived social support as being poor. Lower parental well-being was significantly correlated with poorer family communication, poorer parenting satisfaction, and a greater number of difficulties for the child. Perceived social support was not significantly correlated with parental well-being. A significant difference in parental well-being levels across genders was identified, with female parents having lower well-being levels than males. No significant differences in parental well-being were observed between parents who attended SPACE alone or with a partner, those with and without a history of deliberate self-harm, and those with and without a history of mental health disorders. Parents whose children were not attending school at baseline had significantly lower well-being scores than those whose children were. Parents whose children had received a formal diagnosis of a mental health disorder also had significantly lower well-being scores than those whose children had not.

## Discussion

The aim of the present study was to develop a demographic and psychosocial profile of parents who attended a support programme for parents and carers of adolescents who deliberately self-harm. The most noteworthy aspects to emerge from this profile are discussed hereafter.

Consistent with the findings of previous research on the context of deliberate self-harm of adolescents [16-18], it was found that parental well-being was strongly correlated with the quantity and magnitude of difficulties encountered by the child, as reported by the parent, i.e., parental well-being was lower when child difficulties were greater. These findings may indicate that parents’ mental health is more adversely affected when their child experiences greater difficulties; however, it is also possible that parents whose well-being is lower may be more likely to rate their child’s difficulties as greater, due to their more negative state of mind. Parental well-being itself was relatively poor for the sample at baseline, with 89% of participants meeting criteria for psychological distress. This is congruent with previous investigations of DSH [17,18], although it is not possible to ascertain whether parental distress pre-dated the DSH incident (and thus was a risk factor for DSH in the child), whether it occurred in response to the DSH incident, or whether a combination of both of these situations is occurring. It is also noteworthy that, despite the high rate of poor well-being scores at baseline, roughly half of parents identified themselves as never having a mental health disorder at any point in their lives. This discrepancy could indicate that any mental health issues experienced by parents were not severe enough to be formally diagnosed, or that their issues were severe enough but had never been formally diagnosed.

It was also determined that lower parental satisfaction (relating to satisfaction with oneself as a parent, the parent–child relationship, and the child’s behaviours) and communication problems within the family were similarly associated with lower parental well-being at baseline. These findings are in line with previous research on DSH [13,16,17] and on more general mental health issues [37], which have identified communication problems within the family as being associated with mental health difficulties and poorer family functioning.

The above findings may be associated with the concept of ‘caregiver burden’, whereby existing situational factors and newly-encountered or exacerbated stressors associated with the child’s behaviours combine and contribute towards a decrease in the well-being of the caregiver [20,21]. A number of the findings presented here support the idea that child difficulties relating to DSH may be associated with the deterioration of parental well-being, and the family’s ability to function; for example, parents whose children were not attending school and those whose children had been formally diagnosed with a mental health disorder had significantly lower levels of well-being. The relationships of parental well-being with child difficulties and with family communication may also be an indication of the effects that DSH behaviours may have on a family. This proposition would be supported by previous qualitative investigations of parents’ experiences of adolescent DSH, such as those outlined above, relating to the deleterious effect of DSH on the parent–child relationship and the family structure, as well as the traumatic effect of the child’s DSH behaviours on parents [17-19]. With respect to the content and goals of the SPACE programme, the present findings support the notion that it may be beneficial to provide support to parents and parenting skills training subsequent to DSH episodes, in order to both facilitate better support for the child, and to help parents deal with the challenges that they themselves encounter.

Well-being levels of female parents were found to be lower than those of males. Although the research examining the effects of DSH (and indeed, mental health issues in general) on parents is sparse, a greater impact of child mental health difficulties on females has been reported for parents and carers with respect to schizophrenia [38]. Considering the high proportion of participants in the study who were married or living with a partner (85%), it is unlikely that this gender difference is due to single female parents (who comprised the majority of parents who were not cohabiting) taking on the entire burden of care. It may therefore be useful to examine the balance between parents in terms of the management of the child’s DSH behaviours in future research endeavours, to clarify if females take a greater share of the caregiver burden than males, or if other factors associated with the child’s DSH behaviours impact males and females in unique ways.

It is interesting that parents’ perceived social support was found not to be significantly correlated with well-being, despite 61% of parents rating their social support as being poor. It will be possible to examine in greater detail and report further on the findings related to social support upon the conclusion of the evaluation of the SPACE programme (a randomised controlled trial), wherein the change in perceived social support over time will be examined.

The proportion of parents in the study who were married was surprisingly high at 82%. This is higher than the figure reported for married couples in the general population in the 2011 Irish Census, which was 62.27% [39]. Conversely, the figure for cohabiting couples in the study was 3.8%, which is lower than the 12.96% figure reported for the general population [39]. Despite these differences, the present study’s figures for married and cohabiting couples are similar to those reported for parent programmes for children and adolescents with conduct/behaviour problems in Ireland [40] and Australia [41].

One potential limitation of the study’s design is that parents were not recruited exclusively by referral; in other words, parents who were aware of the SPACE programme could volunteer themselves. While this had the positive effect of broadening the reach of the programme to individuals who would not have otherwise benefited, it also potentially introduces a bias, in that participants who self-selected may have different characteristics to those who refuse to participate or have no interest in participating in group support programmes. This should be taken into account when interpreting the present findings.

## Conclusions

These findings indicate that parents of young people with DSH behaviours face considerable emotional and practical challenges; they have low levels of well-being, parenting satisfaction, social support, and experience poor family communication. Objective factors (e.g., child absence from school) are supported as potentially contributing towards the overall negative effect of DSH behaviours on parents and the family. The present findings also indicate that the influence of DSH behaviours on the perceived social support of parents is unclear, and that further investigation of parents’ perception of the support they receive is required. One natural limitation of the present study is its cross-sectional, survey design, which limits the authors’ ability to determine the direction of the observed relationships outlined above. However, evaluation of the SPACE programme is currently being undertaken by use of a randomised controlled trial (RCT), which is currently nearing completion. Findings from this RCT research should help to clarify and build upon those presented here.

## Competing interests

The authors declare that they have no competing interests.

## Authors’ contributions

SM was involved in the design of the support programme and the RCT, and in the drafting of the manuscript. ER and MN participated in data collection, statistical analysis, and drafting of the manuscript. CB, AC, SC, and JB were involved in the design and running of the support programme. SG participated in statistical analysis. CF was involved in the design of the support programme and the RCT, coordination of the study, and in the drafting of the manuscript. All authors read and approved the final manuscript.

## Supplementary Material

Additional file 1 Background information. Click here for file
