# Supplementary material for: Parents of young people with self-harm or suicidal behaviour who seek help – a psychosocial profile
Source: Child Adolesc Psychiatry Ment Health. 2013 Apr 23;7:13. doi: 10.1186/1753-2000-7-13 (PMC3645953; doi:10.1186/1753-2000-7-13)
Supplement: Additional file 1 — Background information. [file 1753-2000-7-13-S1.doc]

# Appendix A

# BACKGROUND INFORMATION

Thank you for filling out this information, which is anonymous and confidential.

**Your Childs details:**

**Initials…… Age: ……… Nationality…..…………… Attending school** YES/NO **Child’s Gender** MALE/FEMALE

## No of siblings and their position in family (eg. eldest/youngest etc) ………………………………….

**Reason for referral:**

**1.Expression of thoughts of self harm** YES/NO

**2.Episode of Deliberate self harm** YES/NO

If YES which of the following occurred:

**To your knowledge has your child been diagnosed with any of the following?**

| Depression | |  |  |
| --- | --- | --- | --- |
|  |  |  |  |
| Behavioural/Conduct Problems | | |  |
|  |  |  |  |
| Psychosis | |  |  |
|  |  |  |  |
| Eating Disorders | |  |  |
|  |  |  |  |
| ADHD |  |  |  |
|  |  |  |  |
| Alcohol/Drug Misuse | |  |  |

**To your knowledge, when did your child last engage in Deliberate Self Harm?**

| In the past week | |  |
| --- | --- | --- |
|  |  |  |
| 2 to 4 weeks ago | |  |
|  |  |  |
| 1 to 3 months ago | |  |
|  |  |  |
| 4 to 6 months ago | |  |
|  |  |  |
| More than 6 months ago | |  |

**Which of the following Services is your child currently attending?**

| GP Only |  |  |  |
| --- | --- | --- | --- |
|  |  |  |  |
| Counsellor |  |  |  |
|  |  |  |  |
| **Child and Adolescent Mental Health Services:** | | | |
|  |  |  |  |
| Outpatient |  |  |  |
|  |  |  |  |
| Day Hospital |  |  |  |
|  |  |  |  |
| Inpatient Unit |  |  |  |
|  |  |  |  |
| Other (please specify) |  |  |  |

Approximately, how often does your child attend this service?

| Weekly |  |  |
| --- | --- | --- |
| Once every 2-3 weeks | |  |
| Once every month |  |  |
| Once every 3 months |  |  |
| Once every 6 months |  |  |
| Once every year |  |  |

**Your details:**

**
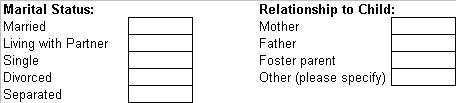
**

**Occupation: _______________________________**

**Have you ever had a problem with any of the following?**

| Depression | |  |
| --- | --- | --- |
| Anxiety |  |  |
| Psychosis | |  |
| Alcohol Problems | |  |
| Self Harm |  |  |

**Many thanks for your help**
